# Impact of the COVID-19 Pandemic on Health Check-ups: A Nationwide Questionnaire Survey in 639 Healthcare Facilities in Japan Society of Ningen Dock

**DOI:** 10.31662/jmaj.2023-0040

**Published:** 2023-06-30

**Authors:** Satoko Yamaguchi, Tomofumi Atarashi, Akira Okada, Shigeru Nasu, Toshimasa Yamauchi, Yasuji Arase, Takao Aizawa, Masaomi Nangaku, Takashi Kadowaki

**Affiliations:** 1 Department of Prevention of Lifestyle-Related Diseases, Graduate School of Medicine, The University of Tokyo, Tokyo, Japan; 2 Japan Society of Ningen Dock, Tokyo, Japan; 3 Medical Check-up Center, JA Hokkaido P.W.F.A.C. Obihiro-Kosei General Hospital, Hokkaido, Japan; 4 Hakuaikai Hospital, Fukuoka, Japan; 5 Department of Diabetes and Metabolism, Graduate School of Medicine, The University of Tokyo, Tokyo, Japan; 6 Health Management Center, Toranomon Hospital, Tokyo, Japan; 7 Aizawa Hospital, Nagano, Japan; 8 Division of Nephrology and Endocrinology, Graduate School of Medicine, The University of Tokyo, Tokyo, Japan; 9 Toranomon Hospital, Tokyo, Japan

**Keywords:** COVID-19 pandemic, health check-ups, cancer screenings, diabetes screenings

## Abstract

**Introduction:**

Health check-ups have been disrupted worldwide by the COVID-19 pandemic, especially at its beginning. In Japan, undergoing annual check-ups is mandatory for full-time employees of all ages, while those other than full-time employees are entitled to undergo nonmandatory cancer screenings and specific health check-ups. To evaluate the impact of the COVID-19 pandemic on health check-ups, we conducted a nationwide questionnaire survey targeting healthcare facilities.

**Methods:**

A questionnaire survey was conducted between December 10, 2021, and January 28, 2022. Healthcare facilities were eligible if they were members of Japan Society of Ningen Dock and could respond via email. The monthly and yearly numbers of examinees undergoing mandatory or nonmandatory check-ups in 2020 and 2021 were compared with those in 2019. The proportions of examinees requiring follow-up visits and adhering to follow-up visits were compared between 2020 and 2019. Precautions taken against COVID-19 were also investigated.

**Results:**

Of the 1,299 eligible facilities, 639 participated (response rate, 49.2%). Health check-up services were suspended in 484 (75.7%) facilities for a median duration of 5 (interquartile range [IQR]: 4-8) weeks. A total of 19,861,230 and 21,748,125 examinees underwent health check-ups in 591 facilities in 2020 and 2021, respectively, 10.0% and 1.4% less than the numbers in 2019. The number of examinees undergoing health check-ups decreased by a median of 8.3% (IQR: −14.6 to −3.1) in 2020 compared to that in 2019, with the largest decrease of 70.3% (IQR −87.9 to −48.5) in May. Although the number of examinees undergoing mandatory check-ups increased in 2021 compared with that in 2019, the number of those undergoing nonmandatory check-ups remained low.

**Conclusions:**

While people eligible for mandatory check-ups were adherent to check-ups in 2021, those ineligible for mandatory check-ups seemed less adherent. Public health efforts to encourage these people to adhere to check-ups during the pandemic are required.

## Introduction

Healthcare services worldwide have been severely affected by the coronavirus disease 2019 (COVID-19) pandemic. Preventive programs, including cancer and diabetes screenings, were disrupted in many countries ^(1), (2), (3)^, raising serious concerns about delays in diagnosis and a subsequent increase in avoidable deaths ^(4), (5), (6), (7)^.

Japan has a universal healthcare insurance system and is actively engaged in promoting nationwide health check-up programs. Most notably, annual health check-ups are mandatory for full-time employees of all ages under the Industrial Safety and Health Act (Supplementary Table 1) ^(8), (9)^. In addition, since 2008, all people aged 40-74 years, regardless of their employment status, are entitled to undergo annual specific health check-ups aimed at screening high-risk populations for lifestyle-related diseases, including hypertension, diabetes, and dyslipidemia ^(10)^. Healthcare professionals offer specific health guidance to those with risk factors for lifestyle-related diseases ^(11)^. Generally, mandatory check-ups based on Industrial Safety and Health Act contain items included in specific health check-ups. Some insurers offer more comprehensive check-ups called “check-ups for prevention of lifestyle-related diseases” or “Ningen Dock,” which contain all items included in the “check-ups based on Industrial Safety and Health Act” and “specific health check-ups,” as well as screenings for certain cancers such as gastric, colorectal, and lung cancers ^(12)^.

Cancer screening is mainly provided by local governments, but it can be included in the aforementioned check-ups provided by the employers or insurers. The national guidelines recommend screenings for gastric, colorectal, lung, breast, and cervical cancer (Supplementary Table 1) ^(13)^.

Health check-ups are not mandatory for those who are not full-time employees, such as those who are self-employed, part-time workers, retired, unemployed, or dependents. However, they are recommended to undergo specific health check-ups and cancer screenings at certain ages. Moreover, they could self-pay for “Ningen Dock.” The types of health check-ups are summarized in Supplementary Table 1.

Although Japan has had a relatively low number of COVID-19-related deaths per population so far, healthcare services have been largely affected by the pandemic, and many of the nonurgent procedures and services have been suspended or delayed ^(14)^. The first state of emergency was declared by the government for 7 prefectures, including Tokyo, on April 7, 2020, which expanded to all 47 prefectures of Japan on April 16. It lasted for 4-7 weeks, depending on the region, until it was lifted for all prefectures on May 25. The Ministry of Health, Labour and Welfare of Japan recommended the suspension of the specific health check-ups and specific health guidance during the first state of emergency ^(15)^. No recommendations to suspend check-ups were issued during the second, third, or fourth states of emergency, which were declared in January, April, and July 2021, respectively. Nevertheless, a decrease in the number of examinees undergoing cancer screenings in 2020 and 2021 was reported ^(16)^. Thus far, the impact of the COVID-19 pandemic on health check-ups remains unclear. Moreover, to the best of our knowledge, no large-scale studies have been conducted on precautions in health check-up settings.

This study aimed to evaluate the impact of the COVID-19 pandemic on health check-ups in Japan by conducting a nationwide questionnaire survey targeting healthcare facilities.

## Materials and Methods

### Questionnaire survey

Healthcare facilities were considered eligible if they were members of Japan Society of Ningen Dock and could respond by email. Japan Society of Ningen Dock was established in 1959 and has approximately 1,700 member facilities including hospitals and clinics across Japan, accounting for around half of all facilities conducting health check-ups ^(12)^. A total of 1,299 facilities that could respond via email were considered qualified for the study. Questionnaires were sent via email on December 10, 2021, and responses were collected by January 28, 2022. The facilities were asked to provide information on their yearly number of examinees between 2017 and 2021, monthly number of examinees between 2019 and 2021, the proportions of examinees requiring follow-up visits and adherence to follow-up visits for cancer and diabetes screenings between 2019 and 2020, and the precautions taken against COVID-19 in each facility. No compensation was given for participation in the survey. The details of the questionnaire are described in Supplementary Method.

### Outcome measures

The rate of change in the number of examinees who underwent check-ups in 2020 or 2021 compared to those in 2019 (pre-COVID-19 year) was calculated for each facility. The cumulative number of examinees between January 2020 and December 2021 was compared to that in 2019. The number of examinees undergoing screenings for cancer, hypertension, diabetes, and dyslipidemia in 2020 among those who underwent comprehensive check-ups (Ningen Dock) was compared to that in 2019. The proportion of examinees requiring follow-up visits among those who underwent screenings and adherence to follow-up visits, defined as the proportion of examinees who attended follow-up visits among those who required them, was calculated for each facility. Further details are provided in Supplementary Method.

### Statistical analysis

Medians and interquartile ranges (IQRs) were used to summarize the continuous variables. Spearman’s rank correlation was used to calculate the correlation coefficients between the number of participating facilities and population of each prefecture and between the median change rates in the number of health check-ups and annual number of COVID-19 cases per population in each prefecture. The numbers of examinees undergoing check-ups in 2019, 2020, and 2021 were compared for each facility using the Friedman test with Bonferroni correction to examine the null hypothesis that there is no difference in the numbers between 2019, 2020, and 2021 for each facility. The number of screenings for cancer, diabetes, hypertension, and dyslipidemia and the proportion of examinees requiring follow-up visits among those who underwent screening and adherence to follow-up visits were compared between 2019 and 2020 for each facility using the Wilcoxon signed-rank test for paired samples, to examine the null hypothesis that the median of the differences between 2019 and 2020 equals zero.

All statistical tests were two-sided, and *P* values < 0.05 were considered significant. All analyses were performed using R v4.1.1. (R Foundation for Statistical Computing, Vienna, Austria).

## Results

Among the 1,299 eligible facilities, 639 responded to the questionnaire survey (response rate: 49.2%). The participating facilities were distributed across all 47 prefectures in Japan, and the number of facilities correlated well with the population of each prefecture (correlation coefficient 0.93; *P* < 0.001; Supplementary Figure 1). Of the participating facilities, 387 (60.6%) were annexed to hospitals, 121 (18.9%) were annexed to clinics, 128 (20.0%) were dedicated to healthcare check-ups, and 3 (0.5%) provided no answer regarding the type of their facilities.

### Changes in the monthly number of examinees undergoing health check-ups

**Figure 1.**
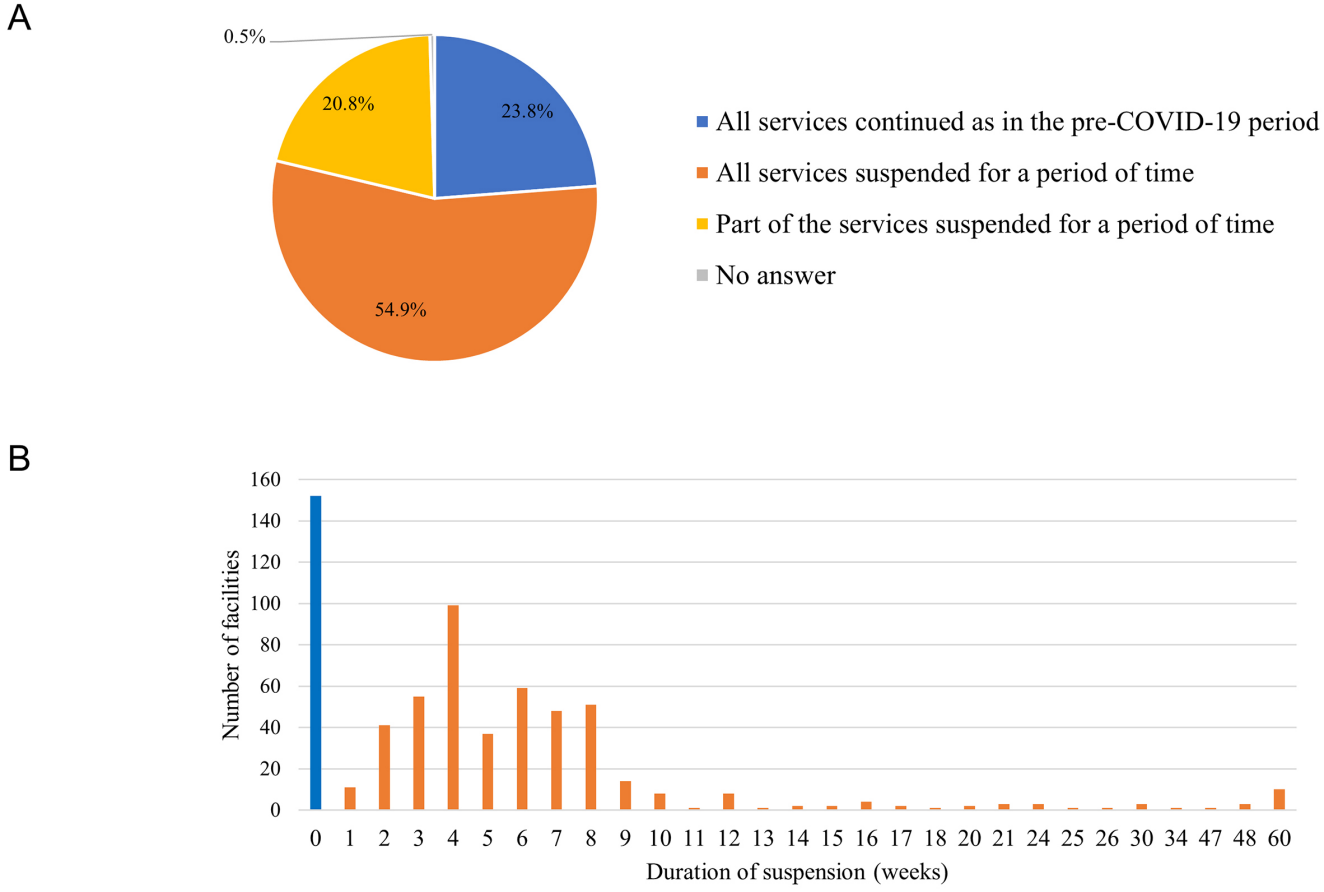
Suspension of health check-up services during the pandemic in healthcare facilities. (A) Facilities were asked whether they suspended all or part of the services (n = 639) (B) Duration of service suspension (n = 624).

**Figure 2.**
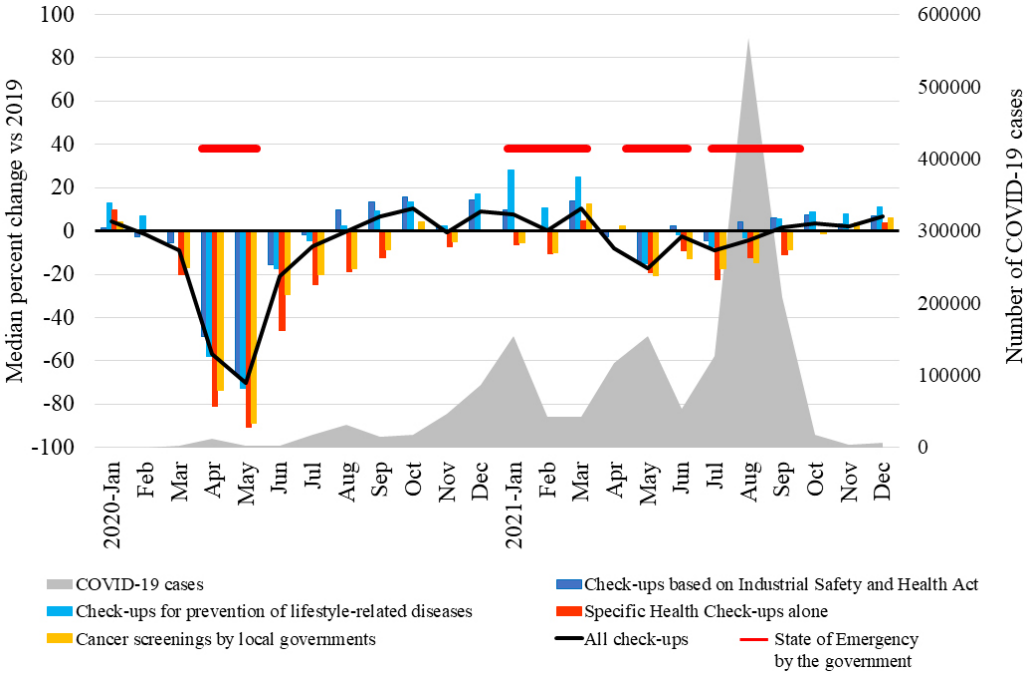
Changes in the monthly number of examinees undergoing health check-ups. Supplementary Table 2 shows the median percentage changes compared to that in the same month in 2019 and IQR

From January 2020 to December 2021, 484 (75.7%) facilities suspended their services for a period of time; among those facilities, 351 (54.9%) suspended all services and 133 (20.8%) suspended part of the services (Figure 1A). The median length of service suspension was 5 weeks (IQR: 4-8 weeks) (Figure 1B). Consistently, the monthly number of examinees undergoing health check-ups changed by a median of −56.7% (IQR: −75.2% to −31.2%) in April and by −70.3% (IQR: −87.9% to −48.5%) in May 2020 compared to those in the same months in 2019 (Figure 2; Supplementary Table 2). The number of examinees undergoing “specific health check-ups alone,” which refer to specific health check-ups for those ineligible for mandatory check-ups, changed by a median of −90.9% (IQR: −100.0% to −62.5%) in May. The number of examinees undergoing cancer screenings by local governments also changed by −89.0% (IQR: −100.0% to −65.6%) in May.

The total monthly number of examinees undergoing health check-ups recovered in August 2020 (median change, −0.1%; IQR: −9.0% to 11.2%; *P* = 0.69) and increased in September and October. Notably, the number of examinees undergoing mandatory “check-ups based on Industrial Safety and Health Act” and “check-ups for prevention of lifestyle-related diseases” increased in January 2021 by a median of 9.8% (IQR: −11.2% to 35.7%; *P* < 0.001) and 28.3% (IQR: 6.2% to 62.8%; *P* < 0.001), respectively, and in March 2021 by a median of 13.8% (IQR: −10.4% to 43.3%; *P* < 0.001) and 24.9% (IQR: 1.1% to 53.4%; *P* < 0.001), respectively, compared to those in the same months in 2019, although the second state of emergency was declared by the government from January 8, 2021 to March 21, 2021 (Figure 2). Furthermore, 52.9% (338/639) of the facilities reported increasing their capacities for check-ups when COVID-19 cases were low, to offer opportunities for catching up.

Pulmonary function tests, gastroendoscopies, specific health guidance for individuals, and specific health guidance for groups were suspended or limited in 88.0% (550/625), 59.2% (348/588), 35.7% (173/484), and 42.3% (44/104) of the facilities that had been offering these services before the pandemic, for a median length of 20 (IQR: 10-21), 2 (IQR: 1-3), 2 (IQR: 1-4), or 2 (IQR: 1-13) months, respectively (Supplementary Figure 2).

### Changes in the annual number of examinees undergoing health check-ups

A total of 19,861,230 and 21,748,125 examinees underwent health check-ups in 591 facilities in 2020 and 2021, respectively. These numbers recorded in 2020 and 2021 were 10.0% and 1.4% lower than those in 2019, respectively. A breakdown of the check-ups is presented in Table 1.

**Table 1.** Changes in the Annual Number of Health Check-Ups in 2020 and 2021 Compared with 2019.

Type of Check-ups	Mandatory Check-ups^a^	Total numbers^b^			Change % for each facility^c^				
		2019 total	2020 total (change%)	2021 total (change%)	2020 median (IQR)	*P* value^d^	2021 median (IQR)	*P* value^d^	Number of facilities
All check-ups	-	22,061,140	19,861,230 (−10.0)	21,748,125 (−1.4)	−8.3 (−14.6, −3.1)	<0.001	−0.5 (−6.0, +4.9)	0.28	591
Check-ups based on Industrial Safety and Health Act	Yes	2,677,734	2,625,750 (−1.9)	2,798,448 (+4.5)	−3.9 (−11.9, +2.7)	<0.001	+1.9 (−6.7, +11.4)	<0.001	531
Check-ups for prevention of lifestyle-related diseases	Yes	2,682,704	2,522,169 (−6.0)	2,807,206 (+4.6)	−4.4 (−12.1, +1.2)	<0.001	+4.5 (−2.7, +11.9)	<0.001	537
Comprehensive check-ups (Ningen Dock)^e^	Yes/No	2,884,244	2,622,728 (−9.1)	2,897,562 (+0.5)	−8.3 (−15.0, −2.7)	<0.001	−0.2 (−7.0, +6.8)	>0.99	563
Specific health check-ups alone^f^	No	590,041	495,615 (−16.0)	548,076 (−7.1)	−14.5 (−26.7, −3.4)	<0.001	−3.8 (−18.1, +9.3)	<0.001	513
Cancer screenings by local governments	No	1,254,749	1,028,044 (−18.1)	1,181,612 (−5.8)	−13.2 (−24.6, 0.0)	<0.001	−3.0 (−16.4, +13.8)	0.007	414
Other in-facility check-ups^g^	No	1,094,981	1,021,032 (−6.8)	1,097,665 (+0.2)	−10.1 (−21.9, +0.9)	<0.001	−3.6 (−17.5, +10.5)	0.008	526
Check-ups using mobile medical vehicles	Yes/No	10,876,687	9,545,892 (−12.2)	10,417,556 (−4.2)	−10.9 (−18.0, −3.9)	<0.001	−3.8 (−13.2, +1.8)	<0.001	192

^a^Whether each type of check-up contains mandatory check-ups (yes), nonmandatory check-ups (no), or both mandatory and nonmandatory check-ups (yes/no) ^b^Total numbers were calculated for 591 facilities ^c^For each type of check-up, the facilities whose annual number of examinees was zero in 2019 were excluded ^d^*P* value comparing the numbers in 2020 and 2021 to those in 2019 using the Friedman test with Bonferroni correction ^e^Comprehensive check-ups (Ningen Dock) as defined by Japan Society of Ningen Dock ^f^“Specific health check-ups alone” refer to specific health check-ups without mandatory check-ups. All items of specific health check-ups are included in the “check-ups based on Industrial Safety and Health Act,” check-ups for prevention of lifestyle-related diseases, and comprehensive check-ups “Ningen Dock” ^g^Includes comprehensive check-ups (Ningen Dock) that do not fulfill the definition of Japan Society of Ningen Dock

**Figure 3.**
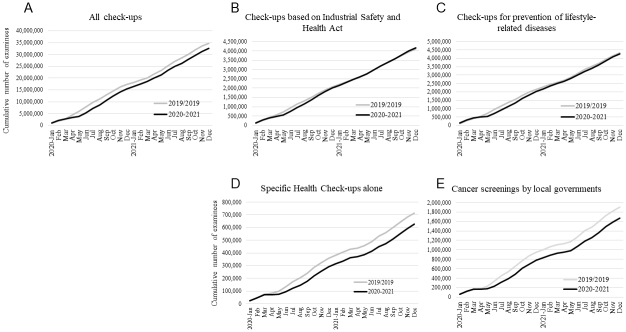
Cumulative numbers of examinees undergoing health check-ups between January 2020 and December 2021. Cumulative numbers of examinees in 2020 and 2021 (black lines) were compared to those in the pre-COVID-19 year (2019) (gray lines) for (A) all check-ups, (B) check-ups based on the Industrial Safety and Health Act, (C) check-ups for the prevention of lifestyle-related diseases, (D) specific health check-ups alone, and (E) cancer screenings by local governments.

The number of examinees undergoing health check-ups in 2020 and 2021 was compared with those in 2019 for each facility. The number of examinees in 2020 changed by a median of −8.3% (IQR: −14.6% to −3.1%; *P* < 0.001) compared to that in 2019, whereas the number in 2021 was comparable to that in 2019, with a median change rate of −0.5% (IQR: −6.0% to 4.9%; *P* = 0.28). For mandatory “check-ups based on Industrial Safety and Health Act” and “check-ups for prevention of lifestyle-related diseases,” the number of examinees changed in 2020 by medians of −3.9% (IQR: −11.9% to 2.7%; *P* < 0.001) and −4.4% (IQR: −12.1% to 1.2%; *P* < 0.001), respectively, whereas this number increased in 2021 by 1.9% (IQR: −6.7% to 11.4%; *P* < 0.001) and 4.5% (IQR: −2.7% to 11.9%; *P* < 0.001), respectively. In contrast, for nonmandatory “specific health check-ups alone” and “cancer screenings by local governments,” compared to 2019, the median change rates in 2020 were −14.5% (IQR: −26.7% to −3.4%; *P* < 0.001) and −13.2% (IQR: −24.6% to 0.0%; *P* < 0.001), respectively, and those in 2021 were −3.8% (IQR: −18.1% to 9.3%; *P* < 0.001) and −3.0% (IQR: −16.4% to 13.8%; *P* = 0.007), respectively (Table 1). Consequently, the cumulative numbers of examinees from 421 facilities for “check-ups based on Industrial Safety and Health Act” and “check-ups for prevention of lifestyle-related disease” decreased from April 2020 but recovered to the pre-COVID-19 level by the end of 2021 (Figure 3B and 3C). By contrast, the difference in the cumulative numbers between 2020/2021 and pre-COVID-19 year (2019) for “specific health check-ups alone” and “cancer screenings by local governments” was increasingly greater by the end of 2021 (Figure 3D and 3E). The cumulative numbers of examinees for all check-ups decreased from April 2020, and the difference in the cumulative numbers between 2020/2021 and pre-COVID-19 year remained nearly constant during the pandemic (Figure 3A).

**Figure 4.**
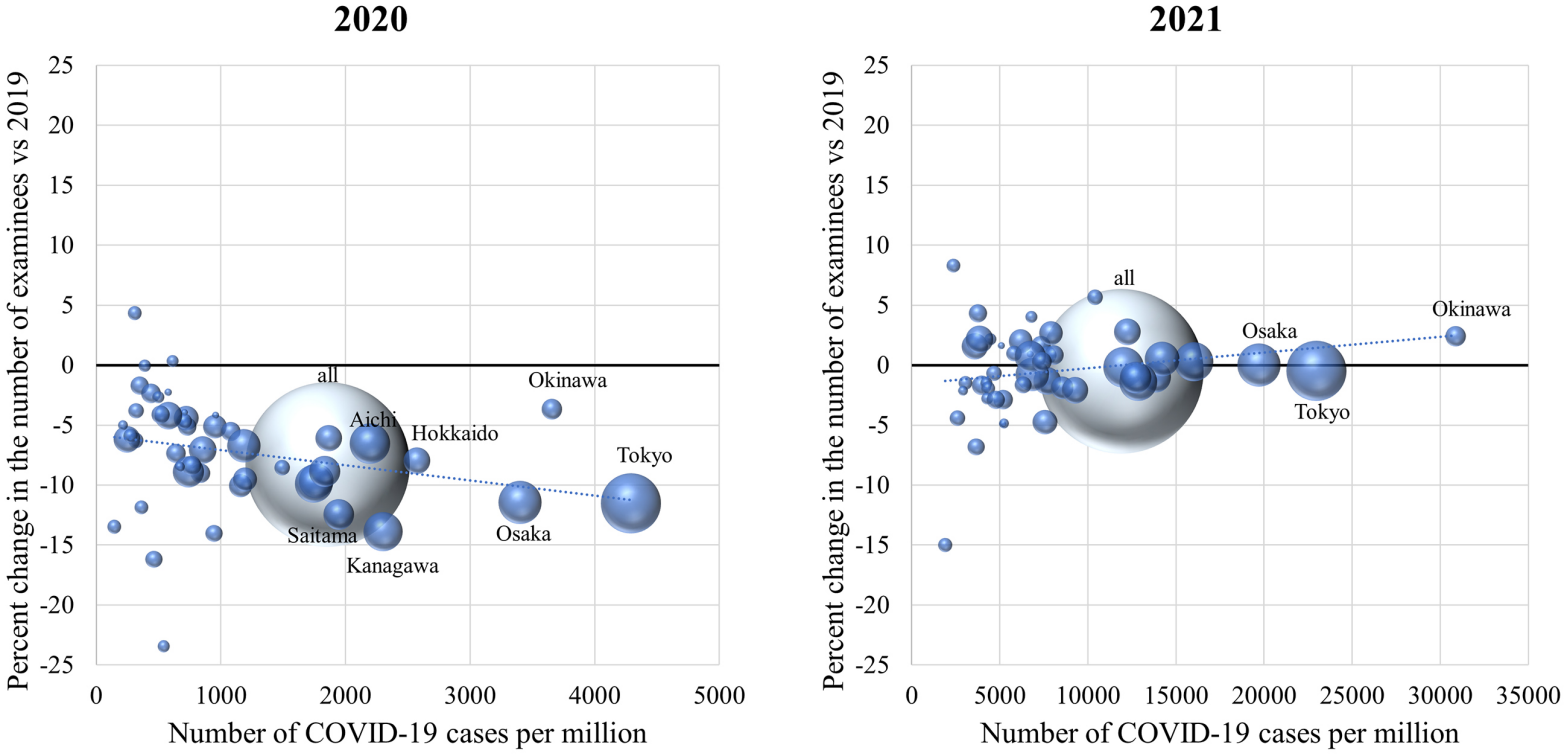
Regional differences in the magnitude of reduction in health check-ups. The magnitudes of reduction in the number of examinees and infection rates of COVID-19 in each prefecture in 2020 (left panel) and 2021 (right panel) are shown. The volume of each sphere represents the numbers of facilities that participated in the survey.

The annual number of COVID-19 cases per population varied among the 47 prefectures, with more than a tenfold difference between the prefectures with the highest and lowest prevalence. The median change rates in the number of health check-ups in 2020 were weakly negatively correlated with the annual number of COVID-19 cases per population in each prefecture (correlation coefficient −0.37; *P* = 0.01) (Figure 4). In contrast, the median changes in 2021 did not correlate with the number of COVID-19 cases (correlation coefficient 0.24; *P* = 0.10) (Figure 4).

### Screenings for cancer, hypertension, diabetes, and dyslipidemia in 2020

The annual number of examinees who underwent screenings for cancer, hypertension, diabetes, and dyslipidemia decreased in 2020 compared to that in 2019 (Table 2). The total number of gastric cancer screenings using endoscopy and contrast radiography decreased by 14.2% and 7.5%, respectively. The number of all other screenings decreased by 8%-10%.

**Table 2.** Changes in the Number of Screenings for Cancer, Diabetes, etc. in 2020 Compared with 2019.

	Total numbers in 2019	Total numbers in 2020 (change %)	Change % for each facility, median (IQR)	*P* value^a^	Number of facilities
Gastric cancer (contrast radiography)	1,183,400	1,095,134 (−7.5)	−7.0 (−15.0, +1.2)	<0.001	366
Gastric cancer (endoscopy)	999,181	856,835 (−14.2)	−9.9 (−19.6, −1.7)	<0.001	360
Colorectal cancer	2,322,061	2,128,041 (−8.4)	−7.8 (−14.4, −3.5)	<0.001	377
Lung cancer	2,519,730	2,293,894 (−9.0)	−8.1 (−14.0, −3.4)	<0.001	377
Breast cancer (mammography)	519,792	472,094 (−9.2)	−8.8 (−15.5, −1.9)	<0.001	370
Breast cancer (ultrasound)	375,503	344,161 (−8.3)	−7.6 (−15.0, +1.8)	<0.001	312
Cervical cancer	660,386	590,999 (−10.5)	−9.5 (−16.8, −2.9)	<0.001	368
Hypertension	2,471,955	2,266,422 (−8.3)	−8.2 (−14.3, −4.1)	<0.001	361
Diabetes	2,442,136	2,239,418 (−8.3)	−8.1 (−14.1, −4.1)	<0.001	360
Dyslipidemia	2,426,457	2,224,723 (−8.3)	−8.1 (−14.2, −4.1)	<0.001	358

^a^*P* value was calculated using the Wilcoxon signed-rank test to examine the null hypothesis that the median of the differences between 2019 and 2020 is zero.

The proportion of examinees requiring follow-up visits among those who underwent screenings in 2020 was compared with that in 2019 for each facility (Table 3). The proportion of examinees requiring follow-up visits decreased for those undergoing screenings for gastric cancer using contrast radiography and endoscopy, lung cancer, and breast cancer using ultrasound, whereas it increased for those undergoing screenings for colorectal cancer, hypertension, diabetes, and dyslipidemia. No significant differences were detected in breast cancer screening by mammography or cervical cancer screening (Table 3).

**Table 3.** Difference in the Proportion of Examinees Requiring Follow-Up Visits between 2020 and 2019.

	Rate % in 2019, median (IQR)	Rate % in 2020, median (IQR)	Difference % (2020-2019), median (IQR)	*P* value^a^	Number of facilities
Gastric cancer (contrast radiography)	3.67 (1.72, 7.31)	3.38 (1.52, 6.30)	−0.33 (−1.33, +0.45)	<0.001^b^	362
Gastric cancer (endoscopy)	3.41 (1.42, 6.26)	3.28 (1.41, 5.97)	−0.19 (−0.85, +0.41)	<0.001^b^	343
Colorectal cancer	5.46 (4.59, 6.46)	5.60 (4.71, 6.50)	+0.06 (−0.41, +0.59)	0.04^c^	371
Lung cancer	1.55 (0.80, 2.60)	1.43 (0.76, 2.49)	−0.06 (−0.43, +0.19)	<0.001^b^	372
Breast cancer (mammography)	4.62 (2.90, 7.03)	4.60 (2.88, 6.92)	−0.08 (−1.38, +0.94)	0.09	363
Breast cancer (ultrasound)	3.00 (1.66, 5.41)	2.66 (1.46, 4.81)	−0.18 (−1.13, +0.54)	0.005^ b^	304
Cervical cancer	1.79 (1.03, 3.27)	1.86 (1.08, 3.43)	+0.03 (−0.50, +0.57)	0.43	362
Hypertension	1.42 (0.49, 2.39)	1.62 (0.57, 2.79)	+0.08 (−0.07, +0.41)	<0.001^c^	350
Diabetes	1.50 (0.71, 2.91)	1.49 (0.70, 3.06)	+0.01 (−0.16, +0.25)	0.047^c^	354
Dyslipidemia	4.26 (1.92, 6.11)	4.45 (2.12, 6.83)	+0.08 (−0.25, +0.70)	<0.001^c^	350

^a^*P* value was calculated using the Wilcoxon signed-rank test for paired samples to examine the null hypothesis that the median of the rate differences between 2019 and 2020 equals zero. ^b^The median of rate differences (rate in 2020 minus rate in 2019 in each facility) was significantly less than zero. ^c^The median of rate differences (rate in 2020 minus rate in 2019 in each facility) was significantly greater than zero.

Adherence to follow-up visits, defined as the proportion of examinees who attended follow-up visits among those who required them, was compared between 2020 and 2019 (Table 4). The adherence to follow-up visits improved in the case of hypertension and diabetes. No significant differences were observed in adherence to follow-up visits for cancer or dyslipidemia between 2020 and 2019 (Table 4).

**Table 4.** Difference in Adherence to Follow-Up Visits between 2020 and 2019.

	Rate % in 2019, median (IQR)	Rate % in 2020, median (IQR)	Difference % (2020-2019), median (IQR)	*P* value^a^	Number of facilities
Gastric cancer (contrast radiography)	57.0 (39.3, 69.8)	57.1 (41.2, 69.0)	−0.6 (−6.5, +6.7)	0.80	305
Gastric cancer (endoscopy)	85.5 (59.6, 98.3)	85.0 (64.6, 98.8)	0.0 (−3.4, +4.6)	0.51	296
Colorectal cancer	54.4 (40.7, 67.1)	54.1 (41.4, 65.2)	−0.1 (−4.6, +4.4)	0.63	325
Lung cancer	70.0 (54.1, 82.2)	69.4 (53.8, 81.8)	+0.4 (−7.1, +8.6)	0.15	329
Breast cancer (mammography)	80.9 (62.6, 90.6)	80.3 (62.3, 89.6)	−0.4 (−6.0, +6.8)	0.81	315
Breast cancer (ultrasound)	76.3 (53.8, 90.9)	78.6 (58.3, 91.7)	0.0 (−6.3, +8.3)	0.26	249
Cervical cancer	70.0 (52.4, 85.7)	71.4 (54.5, 87.6)	0.0 (−9.3, +10.2)	0.38	300
Hypertension	35.2 (19.3, 48.3)	40.0 (22.7, 52.8)	+1.2 (−4.0, +10.9)	<0.001^b^	267
Diabetes	42.6 (22.6, 60.6)	46.2 (27.4, 60.0)	+0.1 (−4.3, +9.5)	0.02^b^	281
Dyslipidemia	33.3 (19.7, 46.3)	35.1 (22.0, 47.0)	0.0 (−3.8, +6.6)	0.12	268

^a^*P* value was calculated using the Wilcoxon signed-rank test to examine the null hypothesis that the median of rate differences between 2019 and 2020 equals zero. ^b^The median of rate differences (rate in 2020 minus rate in 2019) was significantly greater than zero.

### Precautions against COVID-19

Of the 639 facilities surveyed, 322 (50.4%) displayed a poster describing precautions taken against COVID-19 infection in their facilities. These posters informed the examinees that the facilities were following the guideline recommendations to safely perform health check-ups such as wearing masks, handwashing/hand disinfection, checking body temperature and health conditions, good ventilation, preventing overcrowding, and disinfecting high-touch surfaces ^(17)^. When asked whether the facilities had taken specific precautions, 93.6% (598/639) reported making layout changes to secure social distancing, and 66.5% (425/639) reported limiting or rescheduling appointments to prevent overcrowding (Supplementary Figure 3A). Among the free answers provided by 224 facilities on precautions, the most frequent answers were related to checking body temperature and health conditions, disinfection, ventilation, questionnaires including contact and behavior history, installing partitions, installing air purifiers, and wearing personal protective equipment (Supplementary Figure 3B).

When asked about the precautions taken while providing specific health guidance, 242 (50.0%) of the 484 facilities providing specific health guidance answered that no changes were made, 81 (16.7%) answered that they newly introduced online guidance, and 155 (32.0%) answered that they increased the guidance using phone calls or emails (Supplementary Figure 3C).

## Discussion

In this nationwide questionnaire survey that evaluated the impact of COVID-19 on health check-ups in 639 healthcare facilities across Japan, 484 (75.7%) facilities reported suspending the health check-up services completely or partially for a median duration of 5 weeks, the period approximately coinciding with the duration of the first state of emergency lasting 4-7 weeks in April and May 2020. A total of 19,861,230 examinees underwent health check-ups in 591 facilities in 2020, 10.0% less than the number in 2019.

Cancer screening programs were drastically affected at the start of the pandemic ^(18), (19), (20), (21)^. Similarly, more than 60% of the diagnostic tests for diabetes were missed or delayed during the first 6 months of the pandemic in the UK ^(7)^. Although these reports suggest that screening programs were adapted to the COVID-19 pandemic and the numbers recovered within several months, de Jonge et al. estimated that even a 3-month disruption in colorectal cancer screening without a catch-up screening would result in a significant increase in avoidable deaths ^(5)^. Consistent with these previous reports, our results suggest that the overall number of examinees undergoing check-ups sharply declined from April to May 2020, but recovered by August 2020. No apparent decline in the total number of examinees was observed during the second, third, or fourth states of emergency. However, although the number of mandatory check-ups for full-time employees significantly increased in 2021 compared to that in 2019, the number of nonmandatory cancer screenings and specific health check-ups alone remained low in 2021, raising concerns about delays in diagnoses and treatments for cancer and diabetes. People ineligible for mandatory check-ups may have been hesitant to visit healthcare facilities, presumably due to the fear of contracting COVID-19. The public should be well informed that these facilities follow guideline recommendations and make extra efforts to ensure that check-ups are safely performed. In addition, some people may have hesitated to undergo cancer screening for financial reasons, as out-of-pocket payments vary between municipalities ^(13)^. In addition, our results show regional differences in the decline in health check-ups in 2020, with the regions with higher numbers of COVID-19 cases per population being more affected. Previous studies on cancer screening similarly reported that people have been disproportionally affected by the pandemic ^(3), (21)^. Although little has been reported on regional differences within a country, one study reported that the Northeast region experienced the largest decline within the USA, presumably due to surges of COVID-19 cases ^(20)^. The people most impacted by the pandemic likely depend largely on the preexisting healthcare system and infection situation. Public health efforts are necessary to identify these people and remove the barriers.

The proportion of examinees requiring follow-up visits increased among those who underwent screenings for hypertension, diabetes, and dyslipidemia in 2020 compared to that in 2019, which may be a consequence of the reduction in physical activity during the pandemic ^(22)^. Alternatively, this may have been an effect of the changing trends over time, as the proportion of those who were recommended to get specific health guidance among those who underwent specific health check-ups has been gradually increasing since 2016 ^(23)^. The proportion of examinees requiring follow-up visits decreased for gastric cancer screening and increased for colorectal cancer screening, which may reflect the decreasing and increasing incidence rates for gastric and colorectal cancers, respectively, in Japan ^(24)^. The proportion of examinees requiring follow-up visits for lung cancer screening decreased, which may be because not only cancer lesions but also infectious diseases, such as pneumonia, are identified by chest radiography, and respiratory infections decreased in 2020 due to droplet precautions ^(14), (25)^.

The adherence to follow-up visits for hypertension and diabetes improved in 2020 (Table 4). This was unexpected, as a decline was reported in the attendance at scheduled appointments during the pandemic ^(26)^. The improved adherence during the pandemic may be because people with higher health literacy were more inclined to undergo health check-ups even during the pandemic. In addition, it may have been easier to make appointments for clinic visits, as many people were working from home with a flexible schedule in 2020.

### Strength and limitations

The strength of this study lies in it being a large-scale survey targeting facilities across Japan, including 639 facilities that conducted health check-ups for approximately 20,000,000 examinees annually. Moreover, the number of participating facilities in each prefecture correlated well with the population in each prefecture, suggesting that the survey results represent the situation in Japan. Furthermore, a comparison of the different types of health check-ups elucidated important differences in adherence to check-ups. As annual health check-ups are mandatory for full-time employees in Japan, we could identify populations who showed less adherence to check-ups during the pandemic.

This study had some limitations. First, as Japan has a unique health check-up system, the results may not be generalizable to other countries. However, studies from other countries have reported similar results for cancer and diabetes screening programs. Second, adherence to follow-up visits may have been underreported in some facilities. As the Japanese system allows patients to visit any facility of their choice, the facilities rely on the patients to self-report their follow-up visits, and some facilities make more efforts than others to collect reports from patients. However, the adherence was compared between 2020 and 2019 for each facility; therefore, the changes reported were likely to be credible. Third, although three-fourths of the member facilities of Japan Society of Ningen Dock, whose member facilities collectively account for approximately half of the facilities conducting health check-ups in Japan, were eligible for this study, the response rate was relatively low (49.2%). Moreover, it is unknown what percentage of check-ups was covered by the facilities that participated in this study. Nonetheless, as the participating facilities were distributed across all 47 prefectures of Japan and had performed check-ups for approximately 20,000,000 examinees annually, the results likely represent the situation throughout Japan.

### Conclusion

Although the number of examinees undergoing mandatory check-ups recovered in 2021, the number of people undergoing nonmandatory check-ups remained low. It is necessary to encourage people who are ineligible for mandatory check-ups, including those who are self-employed, part-time workers, unemployed, retired, or dependents, to adhere to the check-ups during a pandemic.

The COVID-19 pandemic might have deteriorated public awareness of noncommunicable diseases such as cancer, diabetes, and other chronic conditions. Further studies are warranted to evaluate the long-term impact of the COVID-19 pandemic on preventive programs, including delays in the diagnosis and treatment of cancer and diabetes.

## Article Information

### Conflicts of Interest

SY, AO, and TK are members of the Department of Prevention of Diabetes and Lifestyle-Related Diseases, a cooperative program between The University of Tokyo and Asahi Mutual Life Insurance Company.

### Sources of Funding

This work was supported by MHLW Special Research Program (Grant Number JPMH20CA2046) and MHLW Research Program on Emerging and Reemerging Infectious Diseases (Grant Number JPMH21HA2011). The funding organization has no role in the design of the study, analysis, interpretation of data, or writing the manuscript.

### Acknowledgement

We would like to thank all the facilities that participated in the study for taking the time to respond to the questionnaire. We thank Ms. Emi Yoshikawa and Mr. Akira Nakata of Japan Society of Ningen Dock for their support.

### Author Contributions

SY, TA, AO, SN, MN, and TK designed the study. TA and SN acquired the data. SY analyzed the data. SY and TK wrote the first draft of the manuscript. All authors contributed to interpretation of data and reviewed, revised, and approved the final manuscript. SY and TA contributed equally as first authors. SY and TK contributed equally as corresponding authors.

### Approval by Institutional Review Board (IRB)

The institutional review board of the Graduate School of Medicine at the University of Tokyo (2018030NI) approved this study. This study was performed in accordance with relevant guidelines and regulations (Declaration of Helsinki). The need for informed consent was waived because this was a facility-based survey and did not contain any personal information.

## Supplement

Supplementary Material
