# Supplementary material for: Impact of the COVID-19 Pandemic on Health Check-ups: A Nationwide Questionnaire Survey in 639 Healthcare Facilities in Japan Society of Ningen Dock
Source: JMA J. 2023 Jun 30;6(3):321–31. doi: 10.31662/jmaj.2023-0040 (PMC10407420; doi:10.31662/jmaj.2023-0040)
Supplement: Supplementary Material [file 2433-3298-6-3-0321-s001.pdf]

Impact of the COVID-19 pandemic on health check-ups: A nationwide questionnaire survey in 639 healthcare facilities in Japan Society of Ningen Dock

Supplementary Material

Supplementary Method

Questionnaire survey

The questionnaire consisted of the following three parts: 1) The facilities were asked whether they suspended or limited health check-up services between January 2020 and December 2021, and if so, the types of services that were suspended and the duration of suspension/limitation. Additionally, the facilities were asked about the precautions they took against COVID-19. 2) The facilities were asked to provide the annual number of examinees who underwent health check-ups between 2017 and 2021 according to the type of check-up. Health check-ups were classified into the following types: a) Mandatory check-ups based on Industrial Safety and Health Act for full-time employees; b) Check-ups for prevention of lifestyle-related diseases containing all mandatory items for full-time employees; c) Comprehensive check-ups, ("Ningen Dock") as defined by Japan Society of Ningen Dock, containing all mandatory items for full-time employees; d) Non-mandatory Specific Health Check-ups alone for people aged between 40 and 74 years insured by the National Health Insurance (self-employed, part-time workers, unemployed or retired) or dependents; e) Non-mandatory cancer screenings by local governments; f) Other in-facility check-ups, including comprehensive check-ups (Ningen Dock) that do not fulfill the definition of Japan Society of Ningen Dock; and g) Check-ups using mobile medical vehicles, including both mandatory and non-mandatory check-ups. Additionally, the facilities were asked, if possible, to provide the monthly number of examinees who underwent check-ups between 2019 and 2021. 3) The facilities were asked to provide the number of examinees among those who underwent Ningen Dock in 2019 and 2020 a) who underwent screening for gastric cancer (contrast radiography or endoscopy), colon cancer (fecal occult blood), lung cancer (chest radiography), breast cancer (mammography or ultrasound), hypertension (blood pressure), diabetes mellitus (blood glucose and/or HbA1c), and dyslipidemia (blood lipids; LDL-cholesterol, HDL-cholesterol, triglyceride, and/or total cholesterol and/or non-LDL-cholesterol), b) who required follow-up

visits, except for those who were already being treated at the time of the check-ups, and c) who attended follow-up visits.

Outcome measures

The number of examinees who underwent check-ups in 2020 or 2021 was compared to that in 2019 (pre-COVID year) in total and for each facility (Table 1). The number of “all check-ups” is defined as the sum of the seven types of check-ups described above. The facilities for which the annual number of “all check-ups” was missing in at least one year between 2019 and 2021 (n=2) or whose annual number of “all check-ups” was zero in 2019 (n=46) were excluded, and data from 591 facilities were used in these analyses. For analyses of total numbers in Table 1, the sum of the numbers in 591 facilities for “all check-ups” and for each type of check-up were compared between 2020, 2021, and 2019. For analyses of changes for each facility, the facilities whose annual number of the respective type of check-ups was zero in 2019 were excluded. Then, the median change rates in each type of check-ups were calculated.

Among 591 facilities included in the analysis above, 421 facilities provided the monthly number of examinees. The cumulative numbers of examinees from 421 facilities between January 2020 and December 2021 were compared to those during the pre-COVID-19 year (2019), which were defined as double the numbers between January 2019 and December 2019, because monthly numbers were only available between 2019 and 2021.

The number of examinees undergoing screenings for cancer, hypertension, diabetes, and dyslipidemia in 2020 was compared to that in 2019. The proportion of examinees requiring follow-up visits among those who underwent screenings and adherence to follow-up visits, defined as the proportion of examinees who attended follow-up visits among those who required them, was calculated for each facility. For each screening, facilities whose number of examinees was missing or whose number of examinees was zero in 2019 were excluded from the analyses.

The monthly number of COVID-19 cases in Japan and annual number of COVID-19 cases per population in each prefecture were calculated from the open data of the Ministry of Health, Labour and Welfare and Statistic Bureau of Japan [1, 2].

References

1. Ministry of Health, Labour and Welfare. COVID-19 open data. https://www.mhlw.go.jp/stf/covid-19/open-data.html. Accessed 05 March, 2023.
2. Statistics Bureau of Japan. Overview of Population Projection Results. https://www.stat.go.jp/data/jinsui/2.html. Accessed 05 March, 2023.

Supplementary Table 1. Types of health check-ups in Japan

	(a) Check-ups based on Industrial Safety and Health Act	(b) Check-ups for prevention of lifestyle-related diseases	(c) Comprehensive check-ups "Ningen Dock" defined by the Japan Society of Ningen Dock	(d) Specific Health Check-ups alone*	(e) Cancer screenings by local governments
**Mandatory for examinees (Contained all items defined by Industrial Safety and Health Act)**	○<br>Costs: low	○<br>Costs: moderate	○<br>Costs: high		
**Non-mandatory for examinees**			○<br>(when paid by the examinees)	○	○
**All items of Specific Health Check-ups are included**	○	○	○	○	
**Eligible examinees**	Full-time employees at all ages	Full-time employees aged ≥35 years and insured by the JHIA etc.	Full-time employees insured by certain health insurance society or examinees who wish to pay by themselves	40-74 year old individuals insured by the NHI (self-employed, part-time workers, unemployed, or retired) or dependents of those insured by the JHIA	Residents meeting age criteria
**Costs mainly covered by**	Employers	Insurers	Insurers or examinees	Insurers	Local governments
**Medical interview/physical examination**	●	●	●	●	
**Body weight, BMI, WC**	●	●	●	●	
**Blood pressure**	●	●	●	●	
**Electrocardiography**	●	●	●	□	
**Lipids**	●	●	●	●	
**Liver function tests**	●	●	●	●	
**Blood glucose and/or HbA1c**	●	●	●	●	
**Hemoglobin**	●	●	●	□	
**Urine tests**	●	●	●	●	
**Creatinine**	□	●	●	□	
**Uric acid**		●	●		
**fundoscopy**		□	●	□	
**Eyesight/hearing test**	●	●	●		
**Lung cancer screening (Chest radiography)**	●	●	●		≥40 years old every year
**Stomach cancer screening (Contrast radiography or endoscopy)**		●	●		≥50 years old every two years
**Colorectal cancer screening (fecal occult blood)**		●	●		≥40 years old every year
**Cervical cancer screening (Cervical cytology)**		△	△		≥20 years old every two years
**Breast cancer screening (Mammography)**		△	△		≥40 years old every two years
**Hepatitis B/C virus**		△	△		
**Other items**			Abdominal ultrasound, respiratory function		

			tests		

* (d) Specific Health Check-ups alone refer to Specific Health Check-ups without mandatory check-ups. All items of Specific Health Check-ups are included in (a) the Check-ups based on Industrial Safety and Health Act, (b) Check-ups for prevention of lifestyle-related diseases, and (c) Comprehensive check-ups "Ningen Dock."

JHIA: Japan Health Insurance Association, NHI: National Health Insurance, BMI: body mass index, WC: waist circumference
● essential items ◻ physicians decide if these items are required △optional items

Supplementary Table 2. Changes in the monthly numbers of examinees undergoing health check-ups in 2020 and 2021 compared to those in 2019, as shown in Figure 2

	**All check-ups**	**Check-ups based on Industrial Safety and Health Act**	**Check-ups for prevention of lifestyle-related diseases**	**Specific Health Check-ups alone**	**Cancer screenings by local governments**
**Number of facilities**	421	349	362	242	139
**Month**	Change % for each facility, median (IQR)				
**Jan-2020**	4.2 (-2.0, 12.5)	1.2 (-15.0, 19.5)	13.1 (1.4, 30.3)	9.9 (-14.2, 29.2)	4.1 (-14.0, 32.2)
**Feb**	-1.6 (-7.1, 5.9)	-2.6 (-13.6, 12.0)	7.0 (-2.9, 20.1)	-0.4 (-19.9, 21.2)	0.0 (-17.0, 19.9)
**Mar**	-9.0 (-18.5, -0.3)	-5.7 (-21.9, 8.5)	-0.3 (-14.8, 14.1)	-20.0 (-38.5, -0.2)	-17.0 (-35.2, 12.8)
**Apr**	-56.7 (-75.2, -31.2)	-48.8 (-73.8, -18.7)	-58.2 (-80.5, -25.4)	-81.2 (-100.0, -50.0)	-73.5 (-88.8, -28.9)
**May**	-70.3 (-87.9, -48.5)	-66.4 (-87.8, -35.6)	-72.7 (-95.7, -39.0)	-90.9 (-100.0, -62.5)	-89.0 (-100.0, -65.6)
**Jun**	-20.9 (-35.6, -5.6)	-15.4 (-35.1, 5.0)	-17.6 (-36.3, -1.2)	-46.1 (-67.6, -14.3)	-29.6 (-51.3, -4.5)
**Jul**	-7.1 (-18.4, 2.4)	-1.9 (-16.0, 10.6)	-4.5 (-19.1, 6.2)	-25.0 (-48.7, -2.5)	-20.0 (-42.3, 2.2)
**Aug**	-0.1 (-9.0, 11.2)	9.6 (-4.8, 35.5)	2.2 (-9.9, 17.1)	-18.8 (-40.6, 4.8)	-17.3 (-33.9, 6.1)
**Sep**	6.8 (-1.2, 14.7)	13.3 (0.6, 35.1)	9.4 (-2.2, 24.2)	-12.5 (-39.6, 10.7)	-8.6 (-25.7, 7.7)
**Oct**	10.6 (2.9, 20.3)	15.8 (0.0, 34.1)	13.5 (0.1, 28.2)	0.0 (-20.0, 26.7)	4.2 (-15.0, 25.1)
**Nov**	-0.8 (-7.0, 8.7)	1.7 (-13.4, 17.2)	2.5 (-8.1, 14.8)	-7.5 (-29.2, 16.2)	-4.9 (-24.1, 17.9)
**Dec**	9.1 (0.0, 22.2)	14.4 (-1.8, 37.9)	17.2 (-0.8, 33.2)	0.0 (-21.2, 20.0)	0.0 (-20.7, 17.8)
**Jan-2021**	7.9 (-5.4, 24.5)	9.8 (-11.2, 35.7)	28.3 (6.2, 62.8)	-6.5 (-34.4, 24.8)	-5.6 (-24.6, 24.7)
**Feb**	0.2 (-9.6, 14.3)	-0.2 (-17.5, 21.7)	10.9 (-3.5, 31.4)	-10.5 (-32.0, 19.3)	-10.1 (-28.7, 25.0)
**Mar**	10.5 (-1.7, 24.5)	13.8 (-10.4, 43.3)	24.9 (1.1, 53.4)	4.8 (-19.3, 31.4)	12.5 (-19.1, 42.2)
**Apr**	-8.1 (-18.7, 4.7)	-2.9 (-20.3, 21.2)	0.0 (-19.4, 22.4)	0.0 (-40.0, 58.9)	2.5 (-28.8, 65.8)
**May**	-17.3 (-28.0, -6.2)	-14.3 (-31.0, 5.6)	-15.4 (-28.6, -2.1)	-19.1 (-44.4, 20.6)	-20.7 (-35.6, 4.5)
**Jun**	-2.5 (-12.2, 6.4)	2.4 (-10.0, 26.2)	-1.8 (-14.3, 9.5)	-9.1 (-30.3, 17.8)	-12.8 (-28.7, 12.4)
**Jul**	-9.1 (-17.3, -2.8)	-4.7 (-16.4, 11.6)	-7.1 (-19.9, 3.4)	-22.5 (-41.0, -2.7)	-17.3 (-36.8, 3.4)
**Aug**	-4.1 (-12.9, 6.2)	4.1 (-11.4, 25.5)	-3.3 (-14.3, 10.7)	-12.5 (-31.3, 9.9)	-14.6 (-28.8, 6.1)
**Sep**	1.6 (-7.2, 9.3)	5.9 (-8.9, 25.4)	5.7 (-6.6, 19.3)	-10.8 (-33.7, 10.2)	-8.9 (-27.5, 7.9)
**Oct**	3.5 (-3.1, 11.7)	7.5 (-7.8, 29.0)	9.0 (-1.9, 23.6)	-0.5 (-23.1, 21.9)	-1.1 (-18.6, 19.9)
**Nov**	2.1 (-4.8, 10.6)	3.3 (-12.0, 22.0)	7.8 (-3.6, 20.6)	0.0 (-15.9, 24.4)	3.6 (-12.5, 29.5)
**Dec**	6.9 (-2.6, 18.1)	7.2 (-6.2, 31.8)	11.2 (-1.0, 30.0)	3.9 (-16.1, 33.5)	6.0 (-8.2, 31.3)

Figure Legends

Supplementary Figure 1. Population and number of participating facilities in each prefecture

Supplementary Figure 2. Services that were suspended or limited during the pandemic. The proportions of facilities that suspended or limited services during the pandemic among the facilities that had been offering these services before the pandemic are shown.

Supplementary Figure 3. Precautions against COVID-19 in health check-up facilities. (A) Multiple answer questions (n = 639) and (B) the most frequent free answers about precautions taken to perform health check-ups. (C) Precautions for performing Specific Health Guidance (n = 484).

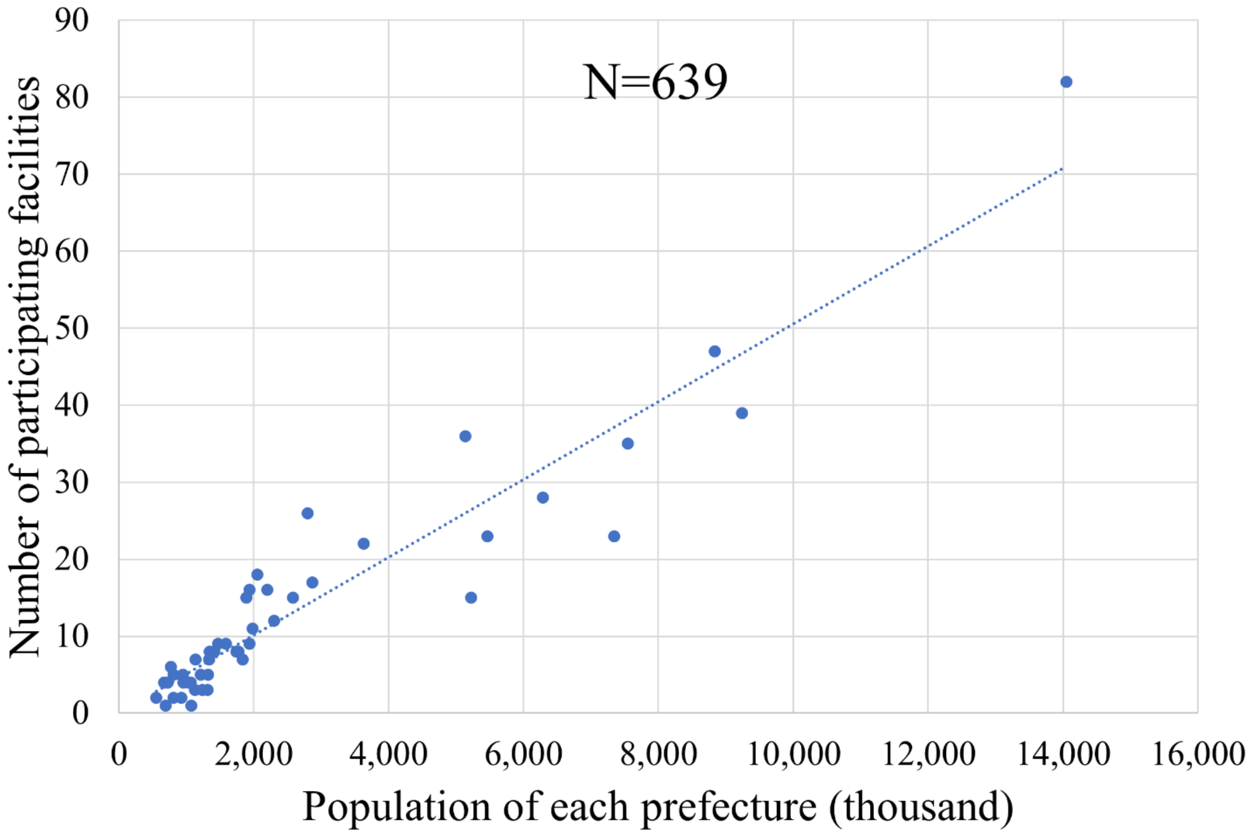

Supplementary Figure 1. Population and number of participating facilities in each prefecture

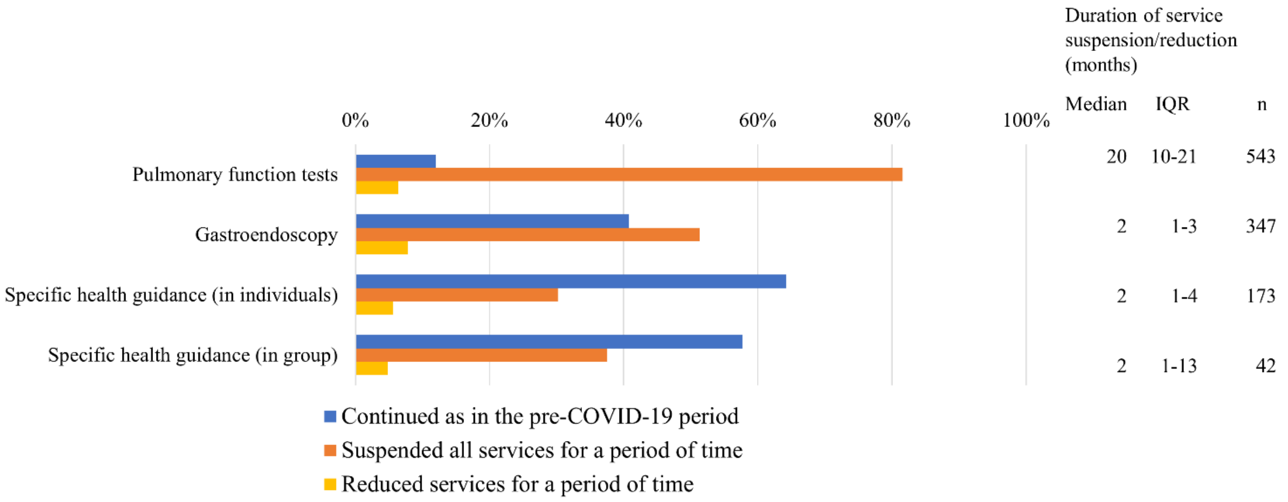

Supplementary Figure 2. Services that were suspended or limited during the pandemic. The proportions of facilities that suspended or limited services during the pandemic among the facilities that had been offering these services before the pandemic are shown.

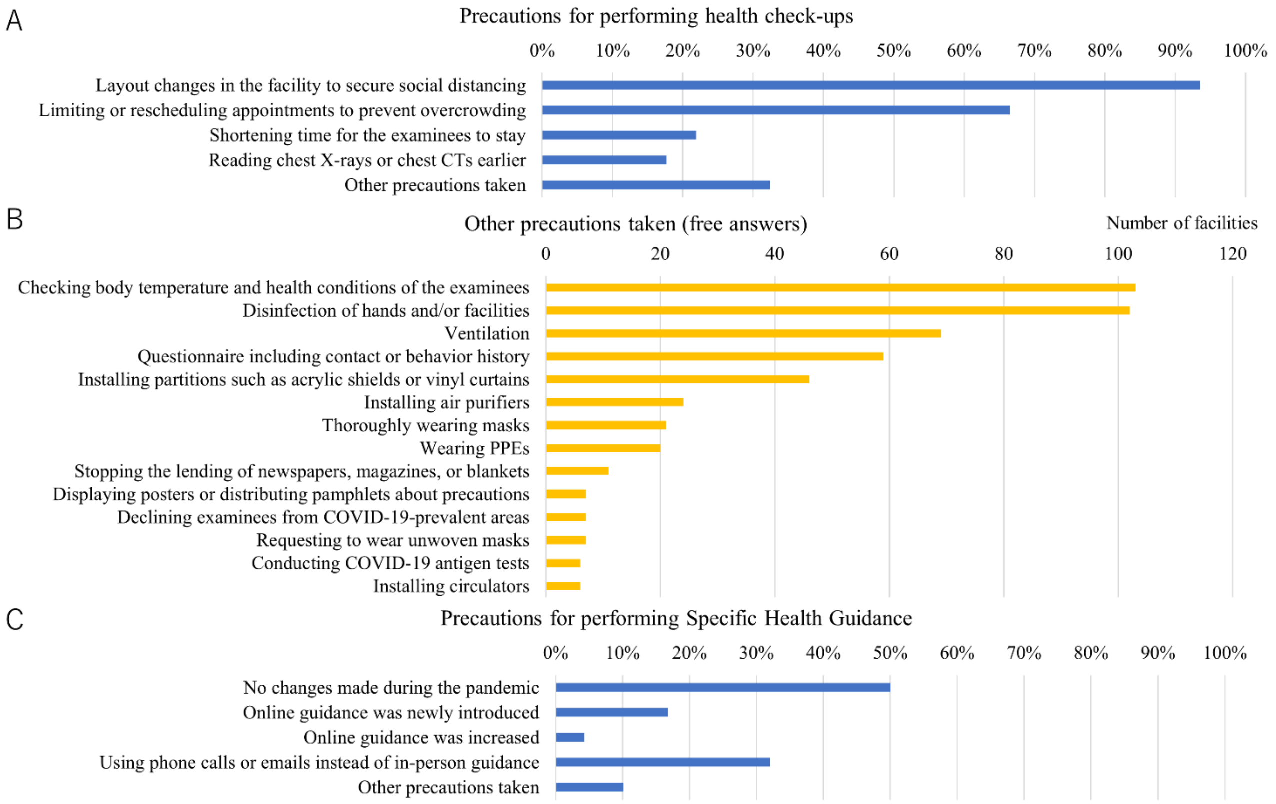

Supplementary Figure 3. Precautions against COVID-19 in health check-up facilities. (A) Multiple answer questions (n = 639) and (B) the most frequent free answers about precautions taken to perform health check-ups. (C) Precautions for performing Specific Health Guidance (n = 484).
